# 基质辅助激光解吸电离-飞行时间质谱法测定*α/β*-二羰基类化合物与标准瓜氨酸化肽段之间的衍生化反应活性

**DOI:** 10.3724/SP.J.1123.2024.02002

**Published:** 2024-07-08

**Authors:** Yanfeng LI, Dandan ZHOU, Xufei CHEN, Juanjuan ZHAO, Chunli GAO, Xingtai QIU, Zichao TANG, Nan DENG, Weining ZHAO, Yangyang BIAN

**Affiliations:** 1.西北大学生命科学学院,陕西 西安 710069; 1. School of Life Sciences, Northwestern University, Xi'an 710069, China; 2.厦门金诺花生物技术有限公司,福建 厦门 361000; 2. Xiamen Jinnuohua Biotechnology Co., Ltd., Xiamen 361000, China; 3.西安交通大学分析测试共享中心,陕西 西安 710049; 3. Analysis and Testing Shared Center of Xi'an Jiaotong University, Xi'an 710049, China; 4.深圳技术大学药学院,广东 深圳 518118; 4. School of Pharmacy, Shenzhen Technology University, Shenzhen 518118, China

**Keywords:** 基质辅助激光解吸电离-飞行时间质谱, 二羰基结构, 瓜氨酸化肽段, 化学衍生, matrix-assisted laser desorption ionization-time-of-flight mass spectrometry (MALDI-TOF MS), dicarbonyl structure, citrullinated peptides, chemical derivatization

## Abstract

蛋白质瓜氨酸化是一种由生物酶调控的不可逆翻译后修饰,其与免疫相关疾病、癌症、神经系统性疾病和老年痴呆症等重大疾病的发生和发展密切相关。蛋白质的瓜氨酸化修饰丰度低、缺乏亲和标签、*m/z*变化小且易受同位素及脱酰胺化作用干扰,因此对蛋白质的瓜氨酸化修饰进行质谱分析面临着巨大挑战。通过对瓜氨酸侧链脲基进行化学衍生反应,可以提高瓜氨酸化肽段的*m/z*差值,引入亲和富集标签,可有效提高瓜氨酸化分析的灵敏度和精准度。本研究以标准瓜氨酸化肽段为研究对象,以基质辅助激光解吸电离-飞行时间质谱(MALDI-TOF MS)为检测手段,对一系列二羰基类化合物与瓜氨酸化肽段之间的化学反应活性进行测定。在对*α*-二羰基类化合物的考察中发现,苯乙二醛、丙酮醛、1,2-环己二酮和1,10-邻二氮杂菲-5,6-二酮对瓜氨酸化肽段的衍生化效率很高,并且能够生成单一的衍生化产物;2,3-丁二酮、乙二醛与瓜氨酸化肽段之间的反应效率也很高,但会生成一系列副反应产物。在对*β*-二羰基类化合物的考察中发现,1,3-环己二酮、2,4-戊二酮在与瓜氨酸化肽段进行反应后,均可以产生单一的衍生化产物,但反应效率很低,不适用于瓜氨酸化肽段的化学衍生反应。实验结果表明,*α*-二羰基结构是实现高效、特异性瓜氨酸化肽段化学衍生反应的基础,*α*-二羰基类化合物中不同的侧链结构又决定了衍生化产物的结构、衍生化效率及副产物的生成。通过进一步合成含有亲和标签的*α*-二羰基类化合物,可以实现瓜氨酸化肽段的特异性富集和精准鉴定,有助于瓜氨酸化蛋白质及其位点鉴定的新方法开发。

## Abstract (en)

Protein citrullination is an irreversible post-translational modification process regulated by peptidylarginine deiminases (PADs) in the presence of Ca^2+^. This process is closely related to the occurrence and development of autoimmune diseases, cancers, neurological disorders, cardiovascular and cerebrovascular diseases, and other major diseases. The analysis of protein citrullination by biomass spectrometry confronts great challenges owing to its low abundance, lack of affinity tags, small mass-to-charge ratio change, and susceptibility to isotopic and deamidation interferences. The methods commonly used to study the protein citrullination mainly involve the chemical derivatization of the urea group of the guanine side chain of the peptide to increase the mass-to-charge ratio difference of the citrullinated peptide. Affinity-enriched labels are then introduced to effectively improve the sensitivity and accuracy of protein citrullination by mass spectrometry. 2,3-Butanedione or phenylglyoxal compounds are often used as derivatization reagents to increase the mass-to-charge ratio difference of the citrullinated peptide, and the resulting derivatives have been observed to contain *α*-dicarbonyl structures. To date, however, no relevant studies on the reactivity of dicarbonyl compounds with citrullinated peptides have been reported. In this study, we determined whether six *α*-dicarbonyl and two *β*-dicarbonyl compounds undergo derivatization reactions with standard citrullinated peptides using matrix-assisted laser desorption ionization-time-of-flight mass spectrometry (MALDI-TOF MS). Among the *α*-dicarbonyl compounds, 2,3-butanedione and glyoxal reacted efficiently with several standard citrullinated peptides, but yielded a series of by-products. Phenylglyoxal, methylglyoxal, 1,2-cyclohexanedione, and 1,10-phenanthroline-5,6-dione also derivated efficiently with standard citrullinated peptides, generating a single derivative. Thus, a new derivatization method that could yield a single derivative was identified. Among the *β*-dicarbonyl compounds, 1,3-cyclohexanedione and 2,4-pentanedione successfully reacted with the standard citrullinated peptides, and generated a single derivative. However, their reaction efficiency was very low, indicating that the *β*-dicarbonyl compounds are unsuitable for the chemical derivatization of citrullinated peptides. The above results indicate that the *α*-dicarbonyl structure is necessary for realizing the efficient and specific chemical derivatization of citrullinated peptides. Moreover, the side chains of the *α*-dicarbonyl structure determine the structure of the derivatives, derivatization efficiency, and generation (or otherwise) of by-products. Therefore, the specific enrichment and precise identification of citrullinated peptides can be achieved by synthesizing *α*-dicarbonyl structured compounds containing affinity tags. The proposed method enables the identification of citrullinated proteins and their modified sites by MS, thereby providing a better understanding of the distribution of citrullinated proteins in different tissues. The findings will be beneficial for studies on the mechanism of action of citrullinated proteins in a variety of diseases.

蛋白质瓜氨酸化是一种由生物酶调控的不可逆翻译后修饰,在生物体的生命活动中发挥着重要调控作用^[1]^。在肽基精氨酸脱亚氨酶(peptidylarginine deiminases, PADs)的催化作用下,蛋白质肽链上的精氨酸会转化为瓜氨酸^[2,3]^(图1a),同时在生理条件下蛋白质丢失一个正电荷,导致其空间构象和功能受到影响^[4]^。最新研究表明,瓜氨酸化修饰不仅参与免疫相关疾病的调控^[5⇓-7]^,与癌症^[8⇓-10]^、中枢神经系统性疾病和老年痴呆症^[11]^等重大疾病也密切相关。例如,在中枢神经系统中,老年痴呆症的发生和病理变化常伴随蛋白质瓜氨酸化的异常表达和PADs的活化,抗瓜氨酸化抗体正在成为老年痴呆症的潜在新型诊断标志物^[12]^。为了全面研究蛋白质瓜氨酸化的功能和调控机理,必须首先确定发生瓜氨酸化的蛋白质种类、位点及丰度,但目前已知的瓜氨酸化蛋白质及其位点是非常有限的。

**图1:**
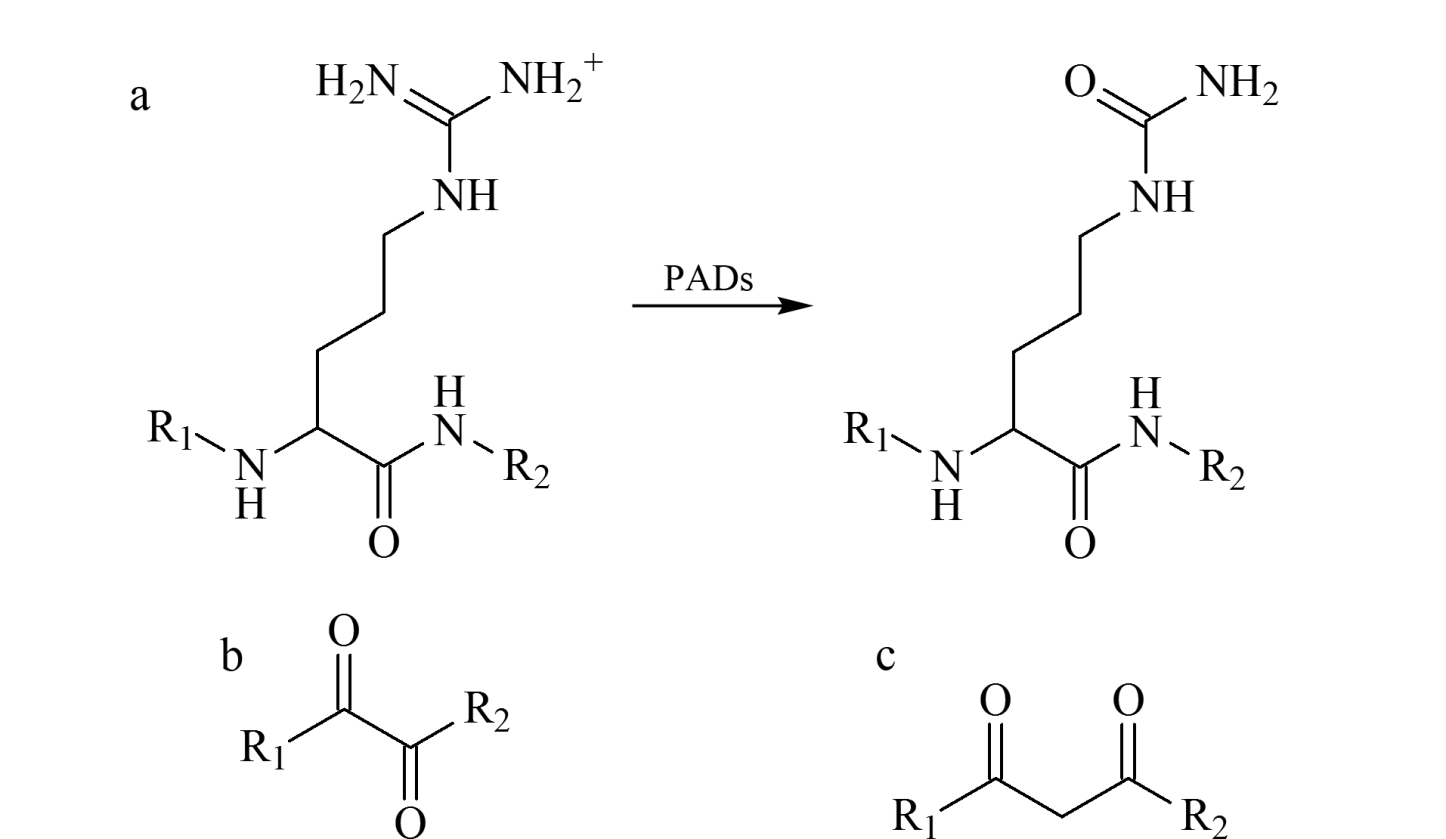
(a)PADs介导的蛋白质瓜氨酸化修饰过程及(b)*α*-二羰基和(c)*β*-二羰基类化合物的结构

生物质谱技术在蛋白质翻译后修饰的研究中发挥着关键作用,它首先利用高分辨质谱仪对翻译后修饰肽段的母离子和碎片离子质量进行测量,再通过数据库匹配,实现肽段的精准鉴定。然而,蛋白质瓜氨酸化修饰的生物质谱分析面临着巨大困难。首先,蛋白质瓜氨酸化的丰度极低,直接进行质谱分析会造成极大困难;其次,瓜氨酸化修饰缺乏亲和标签,无法实现瓜氨酸化肽段的特异性富集;最后,瓜氨酸化修饰的质量差值仅为0.984 Da,极易受到天冬酰胺和谷氨酰胺的脱酰胺化作用干扰以及^13^C等同位素峰的干扰,导致谱图解析的假阳性率极高^[13]^。为解决上述问题,常用的方法是在质谱分析之前对瓜氨酸化肽段进行化学衍生化处理,该方法不仅可以提高瓜氨酸化修饰的*m/z*差值,还可以引入亲和富集标签,能够有效提高瓜氨酸化肽段鉴定的准确度,但通常会存在反应不完全的问题^[14]^。

瓜氨酸侧链上特有的脲基是实现瓜氨酸化肽段特异性化学衍生修饰的理想官能团,已有研究^[15]^将其用于瓜氨酸化肽段的化学衍生反应。早期的研究^[16]^发现,在强酸性条件下,二乙酰单肟(DAMO)可以与瓜氨酸和尿素的胍基发生特异性化学反应,进一步研究发现,DAMO在反应过程中会首先水解为2,3-丁二酮。Holm等^[17]^随后将2,3-丁二酮用于瓜氨酸化肽段的化学衍生反应,所产生的质量差值为50 Da,但得到的衍生化产物稳定性较差;为了提高衍生化产物的稳定性,该团队^[18]^将安替比林和2,3-丁二酮同时用于瓜氨酸化肽段的衍生化反应,衍生化产物的质量差值为238 Da。此外,Li等^[19]^将巯基-生物素和2,3-丁二酮同时用于瓜氨酸化肽段的衍生化反应,可以实现瓜氨酸化肽段的特异性亲和富集。除了2,3-丁二酮,苯乙二醛类化合物也被广泛应用于瓜氨酸化肽段和蛋白质的特异性化学修饰。在强酸性条件下,苯乙二醛^[20]^、罗丹明修饰的苯乙二醛^[21]^、4-叠氮苯基乙二醛^[22]^等均可以与瓜氨酸的脲基发生特异性化学反应。Zhao等^[23]^通过在苯乙二醛的邻位引入硼酸结构,合成了2-乙二醛苯硼酸环单酯化合物,该化合物可以在中性条件下与瓜氨酸的脲基发生特异性化学反应。通过上述研究报道可以发现,在酸性或中性条件下,可用于瓜氨酸化肽段化学衍生反应的化合物均含有*α*-二羰基结构。*α*-二羰基类化合物分子中含有两个相邻的二元酮(图1b),为1,2-二羰基结构,该类化合物的反应活性中心多,是重要的有机合成中间体。在碱性条件下,多种*α*-二羰基类化合物已被应用于胍基化合物的化学衍生反应^[24]^,同时部分含有*β*-二羰基结构的化合物(图1c)也可以与精氨酸的胍基发生反应^[25]^。虽然*α*-二羰基化合物可在不同pH条件下与胍基或脲基发生衍生化反应,但在蛋白质的瓜氨酸化修饰研究中,尚未见关于*α*-二羰基类化合物反应活性的相关研究报道。

基于上述问题,本研究以标准瓜氨酸化肽段为研究对象,以基质辅助激光解吸电离-飞行时间质谱(MALDI-TOF MS)为检测手段,测试了一系列二羰基类化合物与标准瓜氨酸化肽段之间的化学反应活性,为后续瓜氨酸化肽段的亲和富集及蛋白质组学研究等提供了有价值的参考。

## 1 实验部分

### 1.1 仪器、试剂与材料

CP-Light 1000基质辅助激光解吸-飞行时间质谱仪(厦门金诺花科学仪器有限公司);H1750R高速离心机(湖南湘仪实验室仪器开发有限公司);MB102恒温金属浴(杭州博日科技股份有限公司);Smart-S15超纯水仪(上海和泰仪器有限公司)。

标准瓜氨酸化肽段(PL-Cit-AASPFR、AG-Cit-GAGWSLR、PLR-Cit-AASPFR、AGR-Cit-GAGWSLR、biotin-FGG-Cit-GAGHHHHHH,纯度均≥95%)和精氨酸肽段(PL-R-AASPFR,纯度≥95%)均购自国平药业有限公司;*α*-二羰基类化合物(2,3-丁二酮、乙二醛、苯乙二醛、丙酮醛、1,2-环己二酮、1,10-邻二氮杂菲-5,6-二酮)、*β*-二羰基类化合物(1,3-环己二酮、2,4-戊二酮)、三氟甲酸(TFA)、*α*-氰基-4-羟基肉桂酸(CHCA)、乙腈(ACN)和二甲基亚砜(DMSO)均购自西格玛奥德里奇(上海)贸易有限公司。所有实验用水均为超纯水(电阻率为18.2 MΩ·cm)。

### 1.2 标准瓜氨酸化肽段的化学衍生反应

将5条标准瓜氨酸化肽段冻干粉末溶解于超纯水中,分别配制成20 mmol/L的标准瓜氨酸化肽段储备液,分装并保存在-20 ℃冰箱中,使用前用超纯水稀释成1 mmol/L的标准瓜氨酸化肽段溶液。1,10-邻二氮杂菲-5,6-二酮使用80% DMSO水溶液溶解,其余7种二羰基类化合物均使用超纯水溶解,并相应地配制成200 mmol/L的储备液,临用前再分别稀释成50 mmol/L的反应溶液。将5 μL 1 mmol/L的标准瓜氨酸化肽段(以PL-Cit-AASPFR为例)溶液分别与10 μL 50 mmol/L的二羰基类化合物(以2,3-丁二酮为例)溶液进行混合,随后向反应体系中加入25 μL 25%TFA水溶液,再加入10 μL超纯水,使反应体系的总体积为50 μL且pH<1。如未特别指出,所有反应均在37 ℃避光条件下进行,反应时间为2 h。反应结束后,使用超纯水将反应体系稀释20倍,待后续进行MALDI-TOF MS分析。对于其他标准瓜氨酸化肽段和精氨酸肽段,反应条件与上述条件一致。

### 1.3 MALDI-TOF MS分析条件

将CHCA基质溶解于50% ACN水溶液-0.1% TFA水溶液(50∶50, v/v)中,直至基质溶液饱和;将1 μL CHCA基质溶液点在MALDI靶板上,使其在自然条件下晾干,之后将1 μL待测反应体系样品点于MALDI靶板上。质谱分析在CP-Light 1000 MALDI-TOF MS检测系统上完成,实验中的质谱数据均在线性正离子检测模式下获取,脉冲激光波长为355 nm。MALDI-TOF MS的具体参数为高压9000 V、脉冲电压750 V、透镜电压1280 V、延时250 ns、激光能量3.6 ×10^-6^ J、频率60 Hz。质谱仪器质量轴采用实验室配制的标准肽段(RPGFSPFR(*m/z* 962.5086)和YRQSMNNFQGLR(*m/z* 1512.7256))校正液进行质量校正,其中每张质谱图由150个脉冲图谱累加得到。

## 2 结果与讨论

### 2.1 *α*-二羰基类化合物的反应活性

#### 2.1.1 2,3-丁二酮

2,3-丁二酮被广泛应用于脲基化合物(包括尿素^[26]^和瓜氨酸化肽段^[27,28]^)的化学衍生反应。本实验首先以标准瓜氨酸化肽段PL-Cit-AASPFR为研究对象,在强酸、避光条件下与2,3-丁二酮反应2 h。如图2a、2b所示,在经2,3-丁二酮衍生化后的实验组(PL-Cit-AASPFR)中,分别检测到*m/z*为1065.7、1081.4、1131.8、1177.5的多个质谱峰,而在对照组(PL-R-AASPFR,图2c、2d)中未检测到额外的质谱峰,说明衍生化反应发生在瓜氨酸位点(Cit)。此外,由图2a、2b可知,将2,3-丁二酮作为衍生化试剂与PL-Cit-AASPFR进行反应时,会得到一系列副产物。

**图2:**
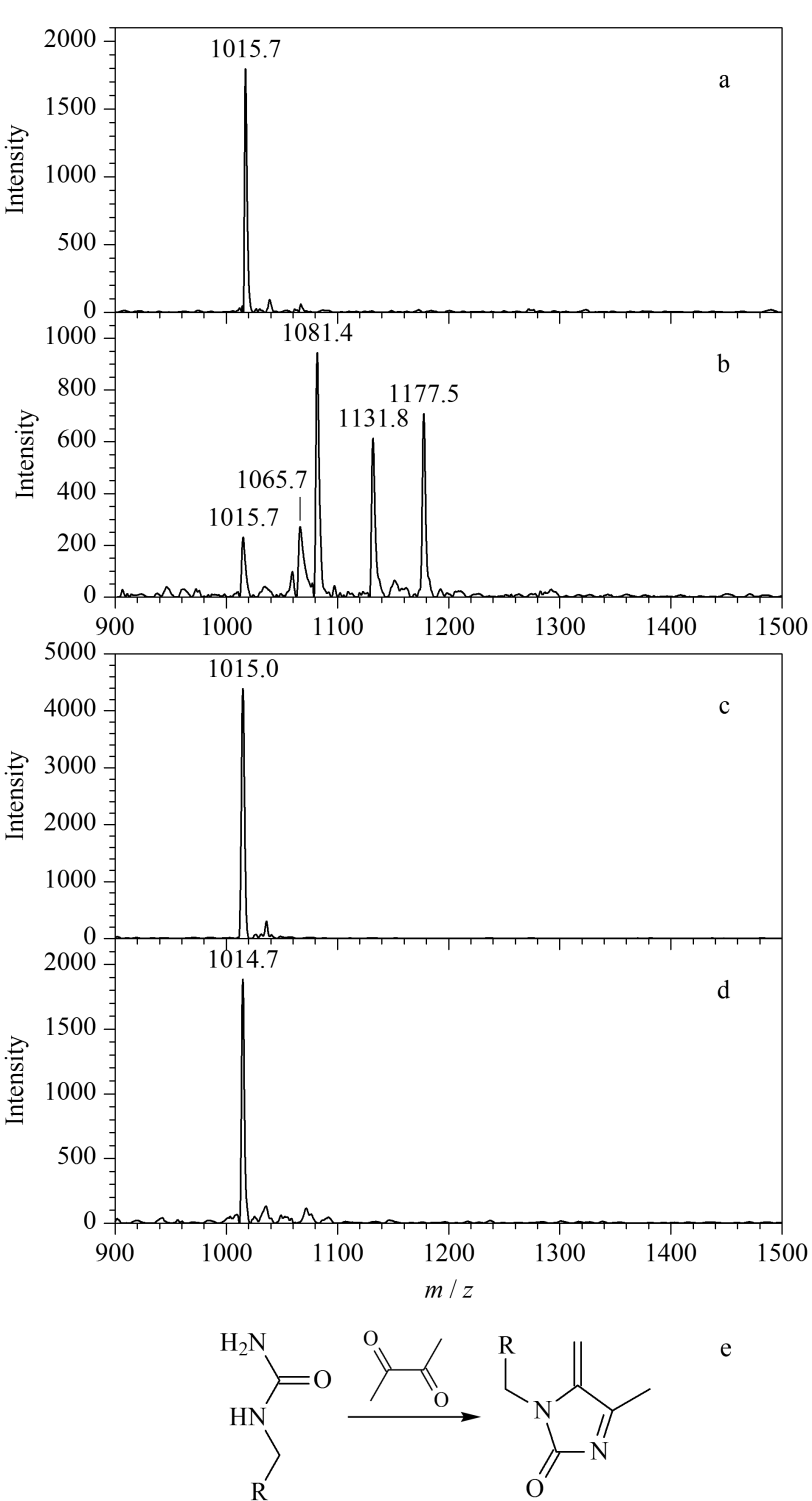
标准肽段PL-Cit-AASPFR、PL-R-AASPFR及其经2,3-丁二酮衍生化后的MALDI-TOF MS图谱及衍生化产物的化学结构

为了测试2,3-丁二酮作为衍生化试剂时的副产物生成现象是否为普遍发生,进一步考察其与另外3种标准瓜氨酸化肽段(PLR-Cit-AASPFR、AG-Cit-GAGWSLR、biotin-FGG-Cit-GAGHHHHHH)的反应情况。如图3所示,与2,3-丁二酮反应后,3种标准瓜氨酸化肽段均产生了*m/z*差值为50和66的质谱峰。与上述结果类似,2006年Holm等^[17]^发现,将2,3-丁二酮与瓜氨酸化肽段(SAVRA-Cit-SSVPGVR)进行反应后,MALDI-TOF MS可检测到*m/z*差值为50、66、116和162 的多个衍生化产物。本研究尝试对衍生化产物的可能化学结构进行推测,但仅能推测出*m/z*差值为50的衍生化产物的可能结构(图2e)。以上结果表明,瓜氨酸化肽段经2,3-丁二酮衍生化处理后,会生成多个衍生化产物。因此2,3-丁二酮不适用于瓜氨酸化肽段的衍生化反应。

**图3:**
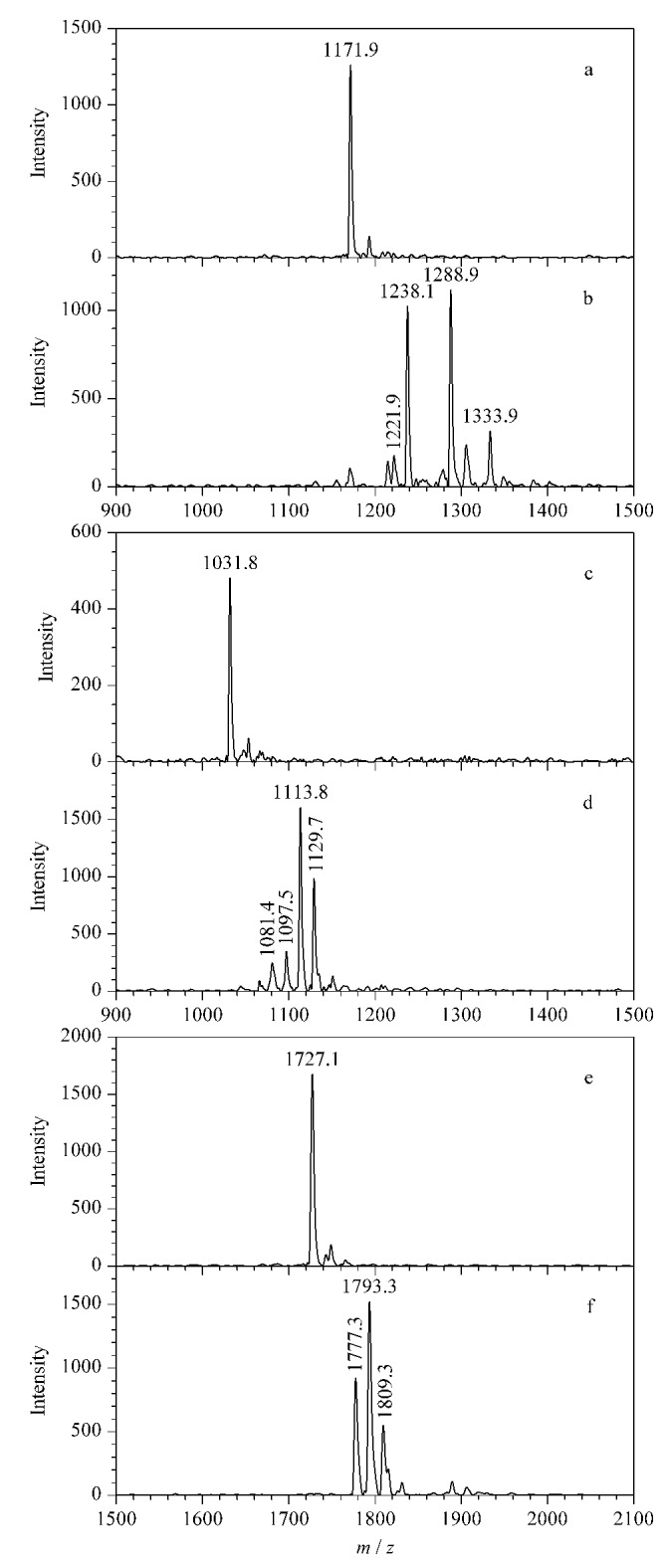
3种标准瓜氨酸化肽段及其经2,3-丁二酮 衍生化后的MALDI-TOF MS图谱

#### 2.1.2 苯乙二醛

据文献[29]报道,苯乙二醛也被广泛应用于瓜氨酸化肽段及蛋白质的特异性修饰,与2,3-丁二酮类似,苯乙二醛同样含有*α*-二羰基结构。本实验以PL-Cit-AASPFR为研究对象,在相同的衍生化条件下对苯乙二醛与PL-Cit-AASPFR之间的反应活性进行测定。结果如图4所示,在反应2 h后,PL-Cit-AASPFR的质谱峰完全消失,而产生了一个新的质谱峰(*m/z* 1131.7),说明苯乙二醛与PL-Cit-AASPFR反应后生成了单一的衍生化产物且反应效率高。由此可见,苯乙二醛能够用于瓜氨酸化肽段的化学衍生反应。

**图4:**
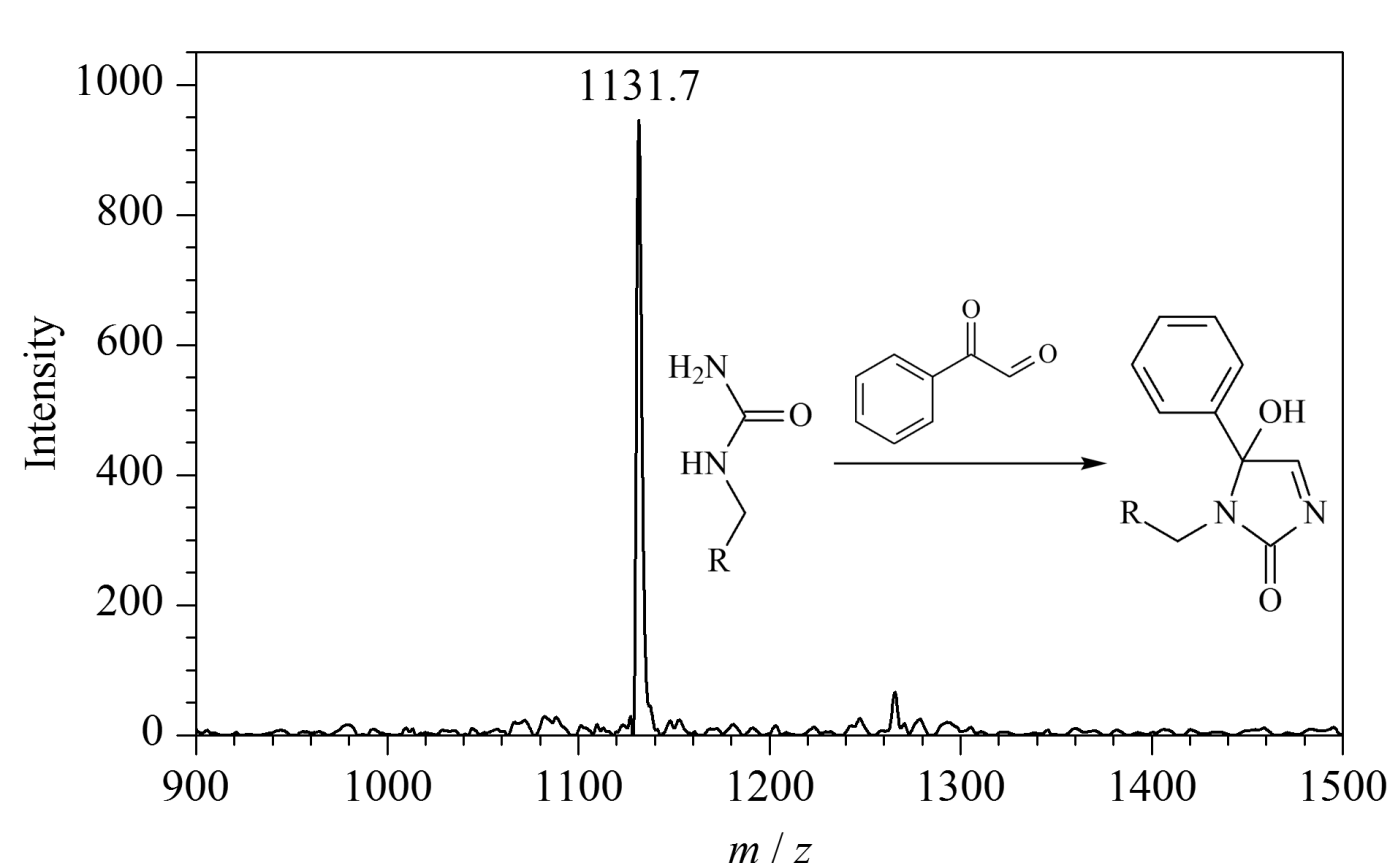
PL-Cit-AASPFR经苯乙二醛衍生化后的 MALDI-TOF MS图谱

#### 2.1.3 乙二醛

乙二醛是结构最简单的、含有*α*-二羰基结构的化合物,本实验对乙二醛与PL-Cit-AASPFR之间的反应情况进行了考察。从图5中可以看到,与乙二醛反应2 h后,PL-Cit-AASPFR产生了*m/z*差值为40、58、116和174的多个质谱峰。已有多个研究^[30,31]^报道发现,将二甲基脲或尿素加入含有乙二醛的酸性或碱性溶液中,在生成4,5-二羟基-2-咪唑啉二酮衍生物的同时,也会生成其他副产物,但副产物的化学结构至今仍不明确。结合文献[30,31]及本研究的实验结果,对PL-Cit-AASPFR衍生化产物的化学结构进行初步推测:*m/z*差值为58的质谱峰可能归属于一个含有4,5-二羟基-2-咪唑啉二酮结构的产物;*m/z*差值为40的产物可能是由上述产物失去1个水分子后得到的,而*m/z*差值为116和174的产物可能是4,5-二羟基-2-咪唑啉二酮结构上邻位二羟基与乙二醛进一步发生偶联反应后的产物。

**图5:**
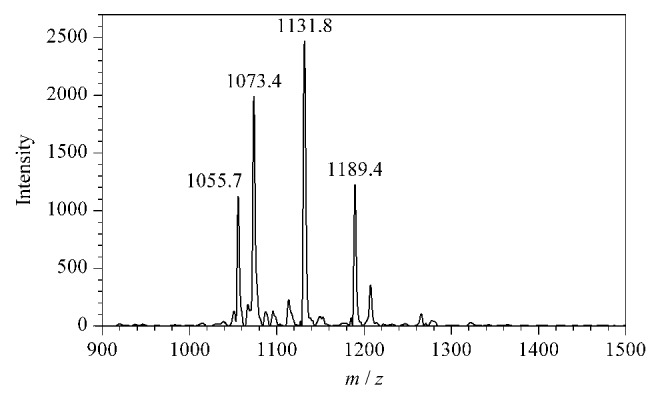
PL-Cit-AASPFR经乙二醛衍生化后的MALDI-TOF MS图谱

为了进一步探究反应时间对乙二醛衍生化效率的影响,将PL-Cit-AASPFR与乙二醛在不同反应时间下进行孵育。如图6所示,在孵育5 min时绝大部分的PL-Cit-AASPFR已完成衍生化反应,但延长反应时间,仍有多个副产物产生,说明乙二醛不适用于标准瓜氨酸肽段的衍生化反应。实验结果表明,通过控制乙二醛的衍生化时间无法避免副产物的产生,原因可能是乙二醛的化学反应活性太高。

**图6:**
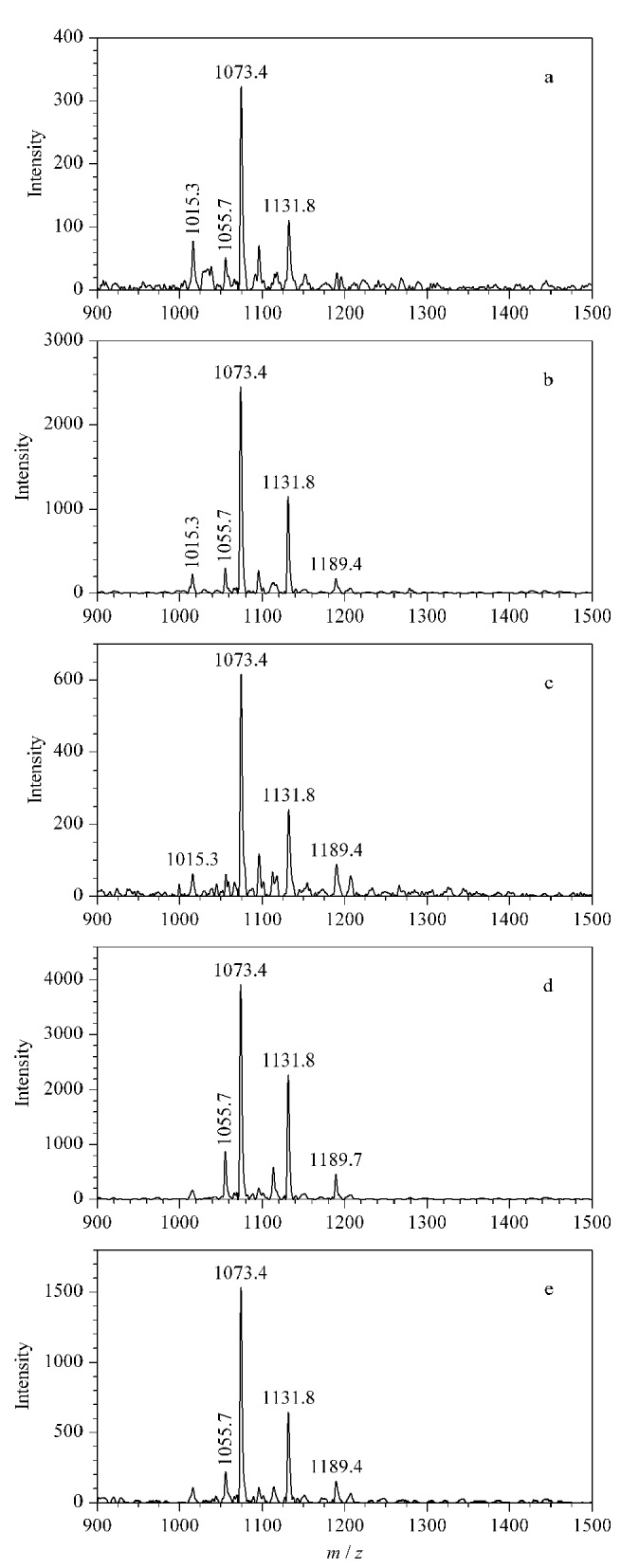
在相同条件下PL-Cit-AASPFR经乙二醛衍生化(a)5、(b)10、(c)20、(d)40和(e)60 min后的MALDI-TOF MS图谱

#### 2.1.4 丙酮醛

进一步测试了丙酮醛与PL-Cit-AASPFR之间的反应活性。结果如图7a所示,与丙酮醛反应2 h后,PL-Cit-AASPFR的质谱峰完全消失,而产生了一个*m/z*差值为54的质谱峰。进一步考察了其他标准瓜氨酸化肽段(PLR-Cit-AASPFR和biotin-FGG-Cit-GAGHHHHHH)与丙酮醛之间的衍生化反应活性,结果如图7b和7c所示,反应2 h后均可检测到一个*m/z*差值为54的质谱峰。上述实验结果说明,丙酮醛与标准瓜氨酸化肽段之间的衍生化反应效率高且衍生化产物单一,适用于瓜氨酸化肽段的化学衍生分析。

**图7:**
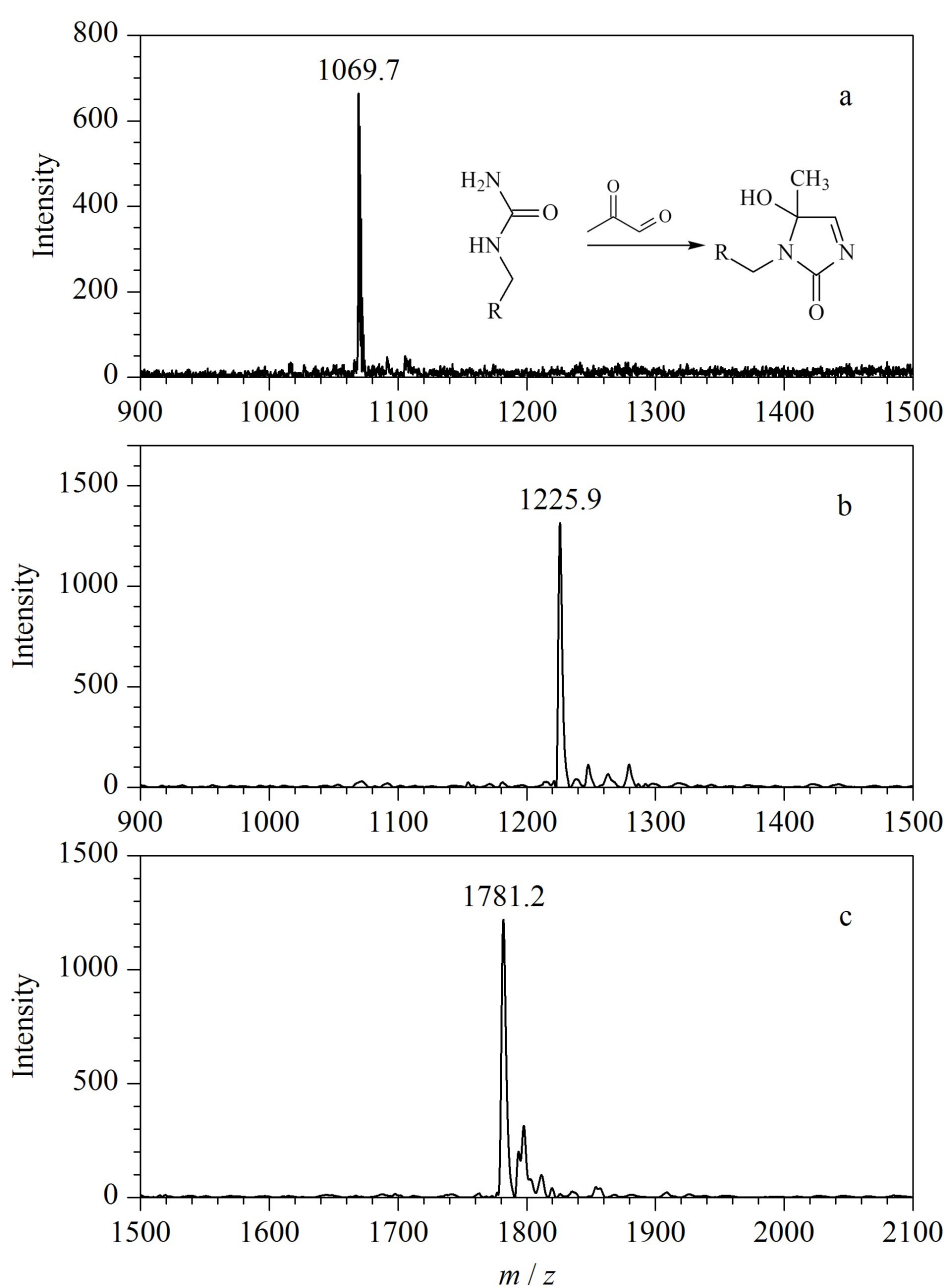
(a) PL-Cit-AASPFR、(b) PLR-Cit-AASPFR、(c) biotin-FGG-Cit-GAGHHHHHH经丙酮醛衍生化后的MALDI-TOF MS图谱

#### 2.1.5 1,2-环己二酮

据文献[31]报道,在碱性溶液中,1,2-环己二酮可以与精氨酸的胍基发生特异性衍生反应,并生成五元环。受上述研究结果的启发,进一步测试了1,2-环己二酮与标准瓜氨酸化肽段之间的衍生化反应活性。如图8所示,与1,2-环己二酮反应2 h后,PL-Cit-AASPFR的质谱峰完全消失,生成了一个*m/z*差值为74的质谱峰。进一步考察其他标准瓜氨酸化肽段(PLR-Cit-AASPFR、AGR-Cit-GAGWSLR和biotin-FGG-Cit-GAGHHHHHH)与1,2-环己二酮之间的反应活性,结果表明,3条标准瓜氨酸化肽段均能反应完全(图9)。上述实验结果说明,1,2-环己二酮可用于瓜氨酸化肽段的特异性化学衍生反应,其反应效率高,并且能够生成*m/z*差值为74的单一衍生化产物。

**图8:**
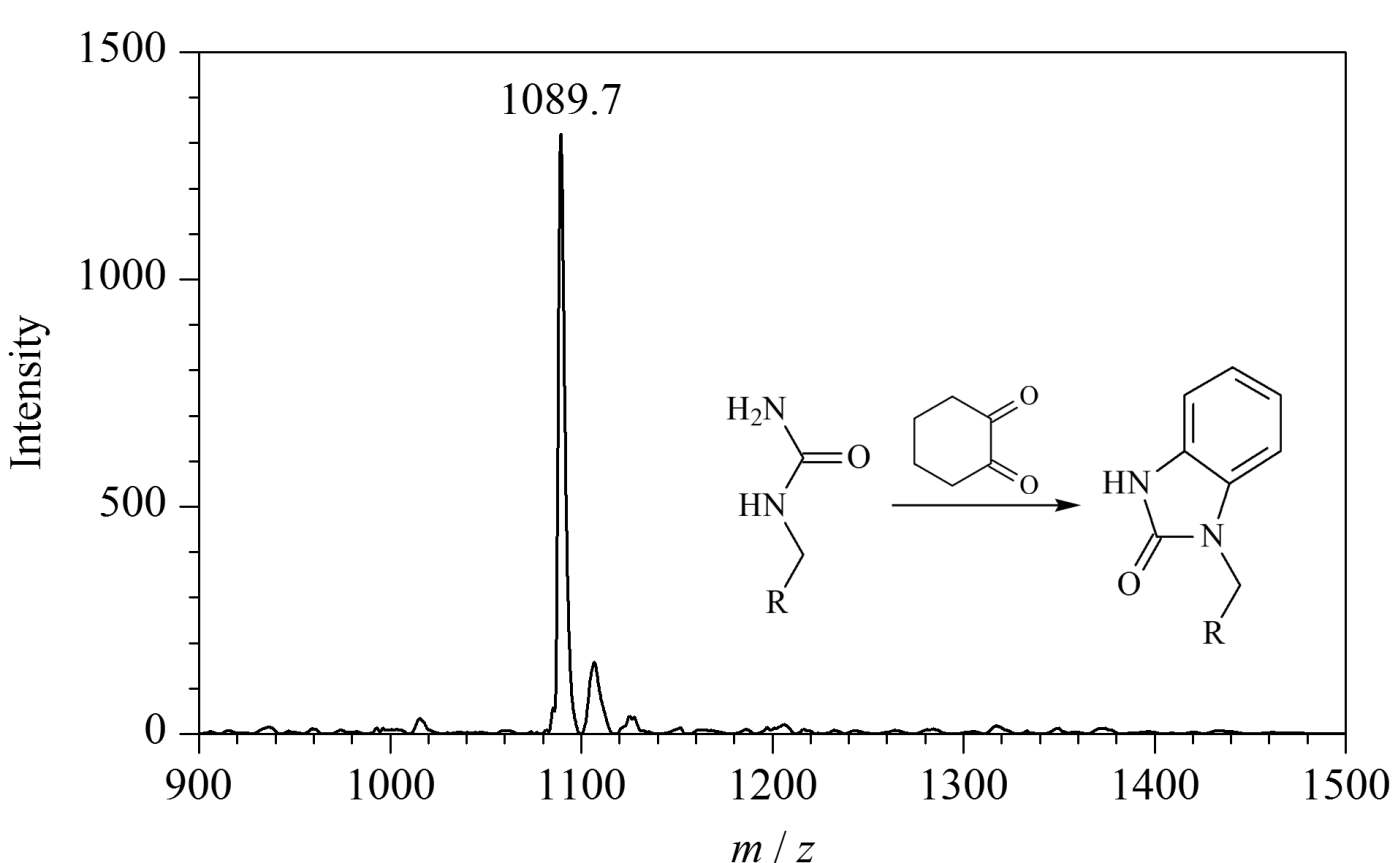
PL-Cit-AASPFR经1,2-环己二酮衍生化后的MALDI-TOF MS图谱

**图9:**
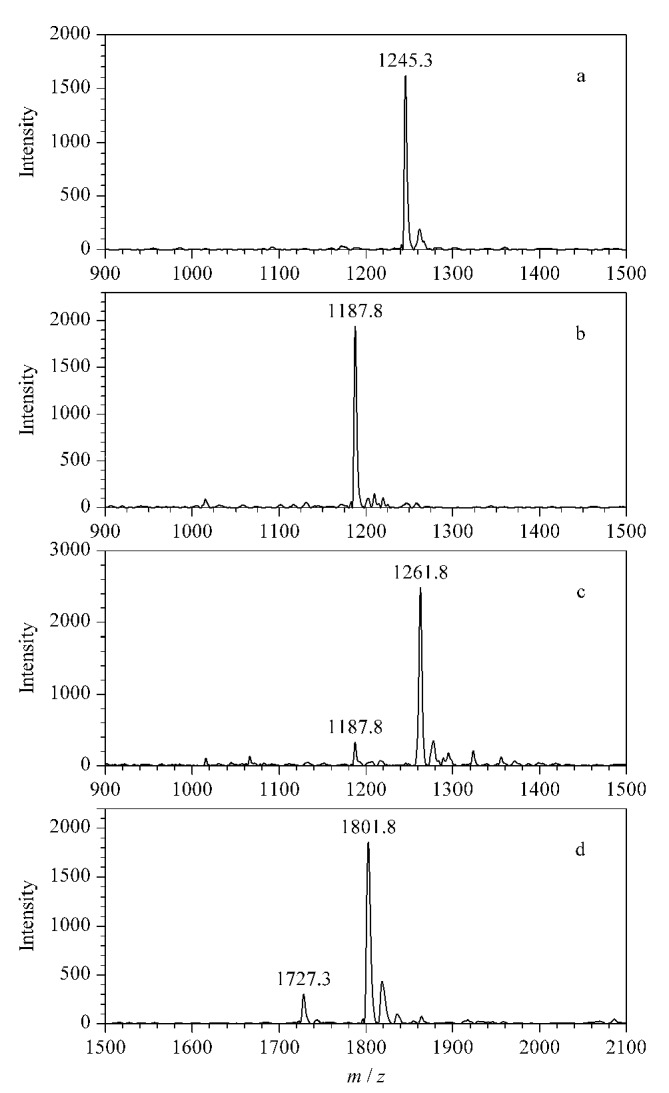
标准瓜氨酸化肽段及经1,2-环己二酮衍生化后的MALDI-TOF MS图谱

#### 2.1.6 1,10-邻二氮杂菲-5,6-二酮

1,10-邻二氮杂菲-5,6-二酮也是一种含有*α*-二羰基结构的化合物,但与1,2-环己二酮相比,其侧链官能团的结构较为复杂。在相同的实验条件下,以PL-Cit-AASPFR为研究对象,考察了1,10-邻二氮杂菲-5,6-二酮与PL-Cit-AASPFR之间的衍生化反应活性。结果如图10所示,反应2 h后仅能检测到*m/z*差值为210的质谱峰,说明二者之间的衍生化反应彻底且产物单一。进一步考察了其他3条标准瓜氨酸化肽段(PLR-Cit-AASPFR、AG-Cit-GAGWSLR和biotin-FGG-Cit-GAGHHHHHH)与1,10-邻二氮杂菲-5,6-二酮之间的反应活性,均得到了单一的衍生化产物(*m/z*差值为210)(图11)。作为一种金属离子螯合剂,邻二氮菲对多种金属离子都具有很强的配位能力。本文用到的1,10-邻二氮杂菲-5,6-二酮具有与邻二氮菲类似的官能团,未来有望使用1,10-邻二氮杂菲-5,6-二酮配位的固定化金属离子亲和材料来实现瓜氨酸化肽段的亲和富集。上述结果说明,虽然1,10-邻二氮杂菲-5,6-二酮的侧链结构较为复杂,但其与瓜氨酸化肽段之间仍具有很高的反应效率且衍生化产物单一,能够用于瓜氨酸化肽段的化学衍生反应。

**图10:**
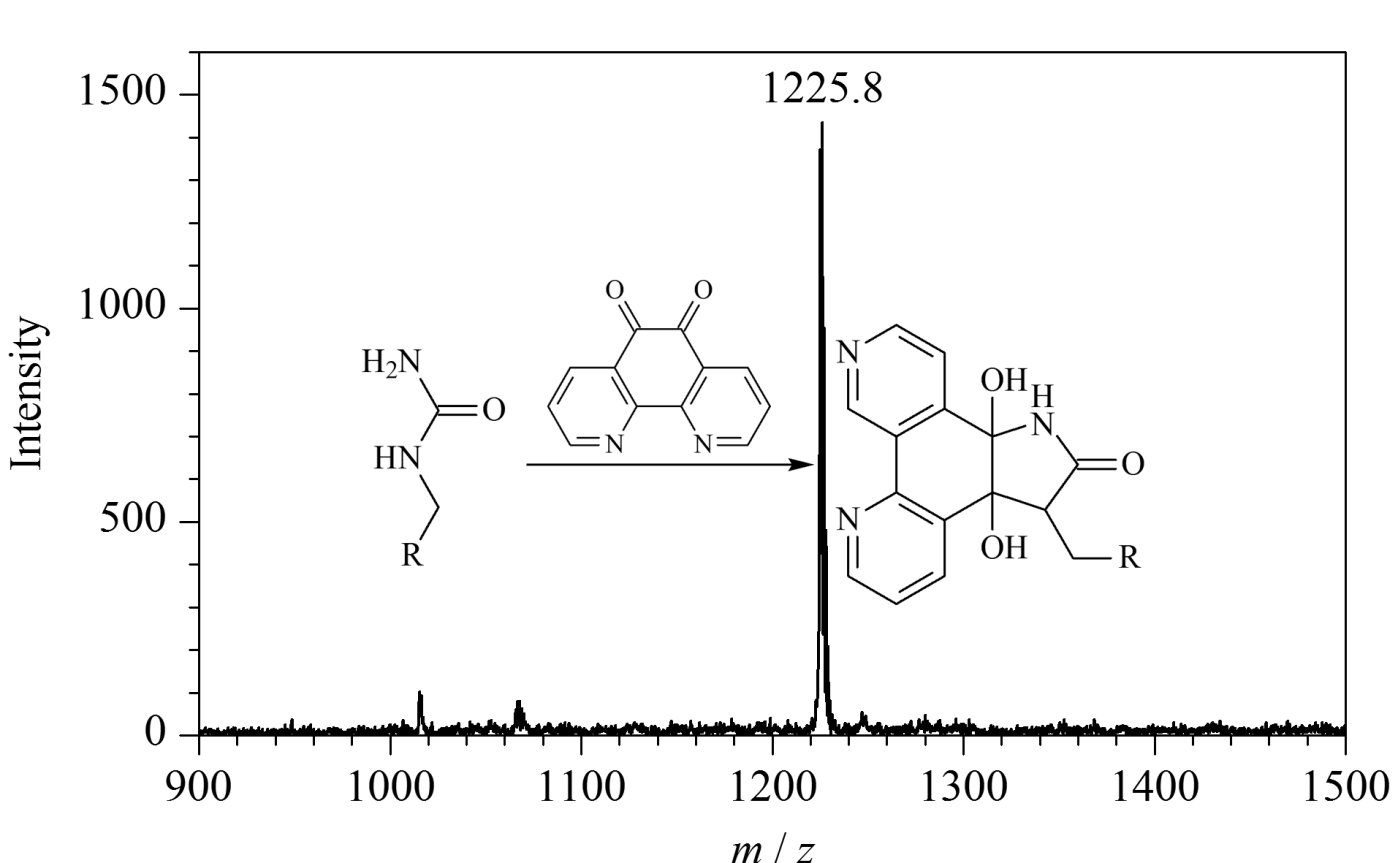
PL-Cit-AASPFR经1,10-邻二氮杂菲-5,6-二酮 衍生化后的MALDI-TOF MS图谱

**图11:**
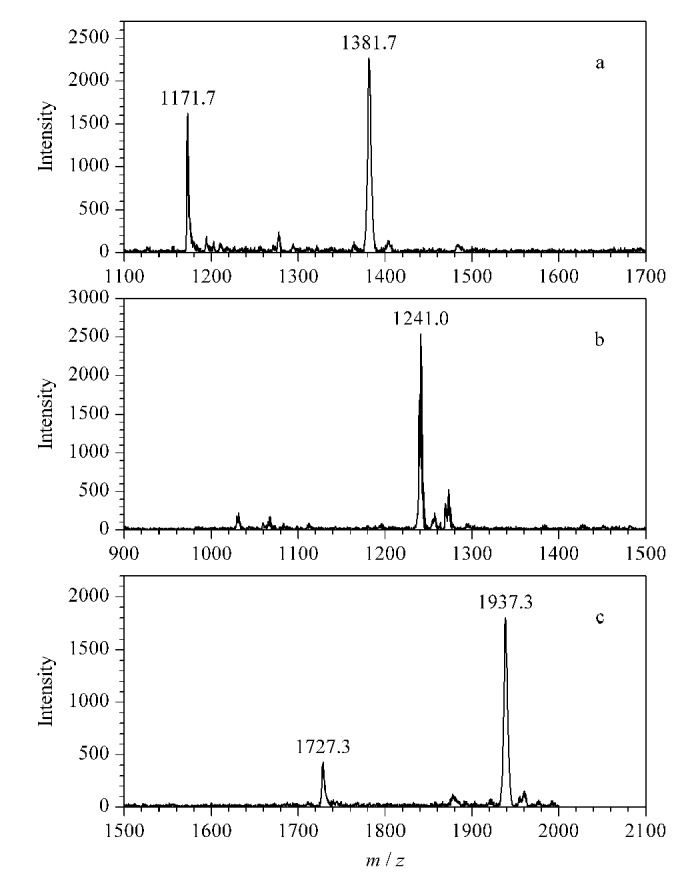
(a) PLR-Cit-AASPFR、(b) AG-Cit-GAGWSLR和(c) biotin-FGG-Cit-GAGHHHHHH经1,10-邻二氮杂菲-5,6-二酮衍生化后的MALDI-TOF MS图谱

### 2.2 *β*-二羰基类化合物的反应活性

以上实验均使用含有*α*-二羰基结构的化合物进行瓜氨酸化肽段的化学衍生反应,据文献[29]报道,部分含有*β*-二羰基结构的化合物(如1,3-环己二酮)也可以与精氨酸的胍基发生反应,从而实现对含有精氨酸的蛋白质和肽段的特异性修饰。受文献[29]启发,本实验进一步测定了两种*β*-二羰基类化合物(1,3-环己二酮和2,4-戊二酮)与PL-Cit-AASPFR之间的反应活性。实验结果表明,在相同的实验条件下反应2 h,PL-Cit-AASPFR均未发生明显的衍生化反应。进一步考察衍生化反应16 h对1,3-环己二酮和2,4-戊二酮衍生化效率的影响。结果发现,在反应16 h后,1,3-环己二酮和2,4-戊二酮均可以与PL-Cit-AASPFR发生特异性衍生化反应,并生成*m/z*差值分别为94和64的衍生化产物,但所得到的反应效率均很低(图12)。上述结果初步说明,含有*β*-二羰基结构的1,3-环己二酮和2,4-戊二酮均不适用于瓜氨酸化肽段的衍生化反应。

**图12:**
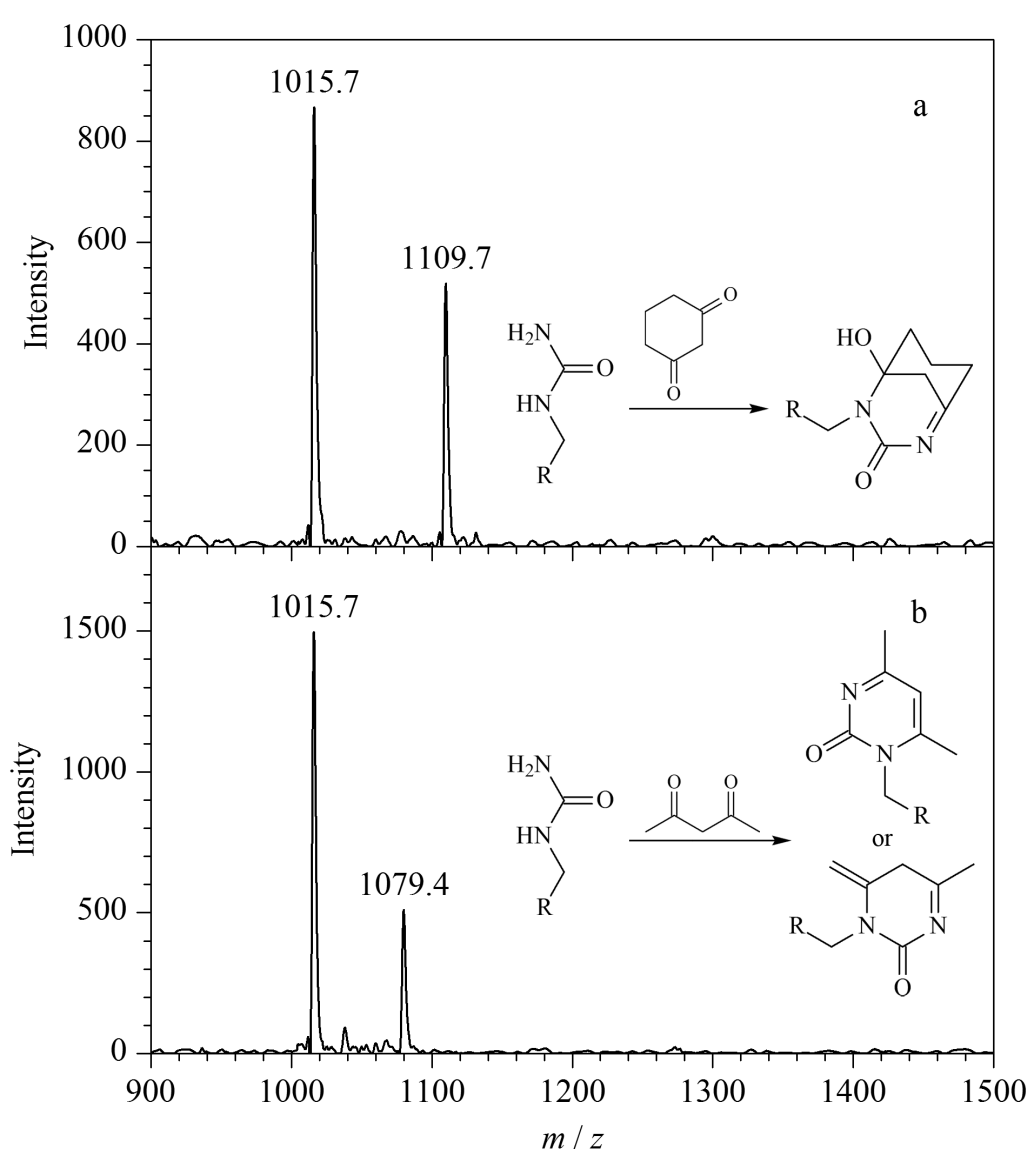
PL-Cit-AASPFR分别经(a)1,3-环己二酮和(b) 2,4-戊 二酮衍生化(16 h)后的MALDI-TOF MS图谱

### 2.3 衍生化反应效率

以PL-Cit-AASPFR为研究对象,统计MALDI-TOF MS图谱中衍生化产物峰面积(*A*)与经衍生化反应后标准瓜氨酸肽段与产物的峰面积之和(*B*),根据*A/B*×100%来粗略计算PL-Cit-AASPFR与二羰基类化合物之间的衍生化反应效率。最后对PL-Cit-AASPFR与不同二羰基类化合物之间的衍生化反应结果进行了总结,结果见表1。

**表1:** 8种二羰基类化合物与标准瓜氨酸化肽段(PL-Cit-AASPFR)之间的衍生化反应总结

Compound	Structure	By- products	Reaction time/h	Reaction efficiency/ %
2,3-Butanedione	*α*-dicarbonyl	√	2	94
Glyoxal	*α*-dicarbonyl	√	2	100
Phenylglyoxal	*α*-dicarbonyl	×	2	100
Methylglyoxal	*α*-dicarbonyl	×	2	100
1,2-Cyclohexanedione	*α*-dicarbonyl	×	2	100
1,10-Phenanthroline-	*α*-dicarbonyl	×	2	100
5,6-dione				
1,3-Cyclohexanedione	*β*-dicarbonyl	×	16	42
2,4-Pentanedione	*β*-dicarbonyl	×	16	35

√: presence of by-products; ×: no by-products.

## 3 结论

本文以标准瓜氨酸化肽段为研究对象,以MALDI-TOF MS为检测手段,考察了一系列二羰基类化合物与标准瓜氨酸化肽段之间的衍生化反应活性。实验结果发现,与*β*-二羰基类化合物相比,*α*-二羰基类化合物与标准瓜氨酸化肽段之间的反应活性更高;此外,部分*α*-二羰基类化合物(如丙酮醛和1,10-邻二氮杂菲-5,6-二酮)不仅可以用于瓜氨酸化肽段的特异性化学修饰,还有望应用于瓜氨酸化肽段的亲和富集以及后续的蛋白质组学研究。本工作为蛋白质瓜氨酸化修饰在自身免疫缺陷等疾病中的作用研究方面提供了技术支持。

## References

[1] Alghamdi M, Al Ghamdi K A, Khan R H, et al. Cell Mol Life Sci, 2019, 76(23): 4635 31342121 10.1007/s00018-019-03237-8 PMC11105357

[2] Mondal S, Wang S, Zheng Y, et al. Nat Commun, 2021, 12(1): 45 33398026 10.1038/s41467-020-20279-w PMC7782748

[3] Sun B, Tomita B, Salinger A, et al. Matrix Biol, 2021, 102: 70 34274450 10.1016/j.matbio.2021.07.001 PMC8502204

[4] Ciesielski O, Biesiekierska M, Panthu B, et al. Cell Mol Life Sci, 2022, 79(2): 94 35079870 10.1007/s00018-022-04126-3 PMC8788905

[5] Umeda N, Matsumoto I, Kawaguchi H, et al. Clin Rheumatol, 2016, 35(5): 1181 26415740 10.1007/s10067-015-3082-z

[6] Gordon R A, Herter J M, Rosetti F, et al. JCI Insight, 2017, 2(10): e92926 28515361 10.1172/jci.insight.92926 PMC5436537

[7] Hanata N, Shoda H, Hatano H, et al. Front Immunol, 2020, 11: 1095 32655553 10.3389/fimmu.2020.01095 PMC7324481

[8] Gu W, Zhang M, Gao F, et al. Int Immunopharmacol, 2022, 110: 108965 35764017 10.1016/j.intimp.2022.108965

[9] Luo X, Chang S, Xiao S, et al. Neoplasia, 2022, 33: 100835 36113195 10.1016/j.neo.2022.100835 PMC9483803

[10] Zhu D, Lu Y, Wang Y, et al. Pharmaceutics, 2022, 14(11): 2414 36365233 10.3390/pharmaceutics14112414 PMC9699117

[11] Wang L L, Song Y P, Mi J H, et al. Med Hypotheses, 2021, 146: 110466 33412502 10.1016/j.mehy.2020.110466

[12] Mukherjee S, Perez K A, Dubois C, et al. ACS Chem Neurosci. 2021, 12(19): 3719 34519476 10.1021/acschemneuro.1c00474

[13] Clancy K W, Weerapana E, Thompson P R. Curr Opin Chem Biol, 2016, 30: 1 26517730 10.1016/j.cbpa.2015.10.014 PMC4731267

[14] Wang B, Fields L, Li L. Proteomics, 2023, 23(21/22): 2200286 10.1002/pmic.202200286 PMC10285031 36546832

[15] Moelants EA, Van Damme J, Proost P. PLoS One, 2011, 6(12): e28976 22194966 10.1371/journal.pone.0028976 PMC3241686

[16] Zarabian B, Koushesh F, Vassef A. Anal Biochem, 1987, 166(1): 113 3118740 10.1016/0003-2697(87)90553-7

[17] Holm A, Rise F, Sessler N, et al. Anal Biochem, 2006, 352(1): 68 16540076 10.1016/j.ab.2006.02.007

[18] Stensland M, Holm A, Kiehne A, et al. Rapid Commun Mass Spectrom, 2009, 23(17): 2754 19639564 10.1002/rcm.4185

[19] Li Z, Wang B, Yu Q, et al. Anal Chem, 2022, 94(7): 3074 35129972 10.1021/acs.analchem.1c04073 PMC9055876

[20] Bicker K L, Subramanian V, Chumanevich A A, et al. J Am Chem Soc, 2012, 134(41): 17015 23030787 10.1021/ja308871v PMC3572846

[21] Tutturen A E V, Holm A, Jørgensen M, et al. Anal Biochem, 2010, 403(1): 43 20399192 10.1016/j.ab.2010.04.012

[22] Hensen S M M, Boelens W C, Bonger K M, et al. Molecules, 2015, 20(4): 6592 25875038 10.3390/molecules20046592 PMC6272700

[23] Zhao H, Shan A, Liang Y, et al. Org Lett, 2022, 24(34): 6351 35997298 10.1021/acs.orglett.2c02722

[24] Shih C T, Kuo B H, Tsai C Y, et al. Org Lett, 2022, 24(25): 4694 35727008 10.1021/acs.orglett.2c01970

[25] Wang Q, Li Z, Zhang S, et al. Proc Natl Acad Sci U S A, 2022, 119(43): e2205255119 10.1073/pnas.2205255119 PMC9618127 36256816

[26] Goos R J, Abdraimova N, Johnson B E. Commun Soil Sci Plant Anal, 2015, 46(4): 424

[27] Wang B, Li Z, Shi Y, et al. Mass Anal Chem, 2024, 96(6): 2309 38285917 10.1021/acs.analchem.3c02646 PMC11526168

[28] Tilvawala R, Thompson P R. Curr Opin Struct Biol, 2019, 59: 205 30833201 10.1016/j.sbi.2019.01.024 PMC6717693

[29] Bicker K L, Subramanian V, Chumanevich A A, et al. J Am Chem Soc, 2012, 134(41): 17015 23030787 10.1021/ja308871v PMC3572846

[30] Vail S L, Barker R H, Mennitt P G. J Org Chem, 1965, 30(7): 2179

[31] Ghandi M, Olyaei A, Salimi F. Molecules, 2006, 11(10): 768 17971753 10.3390/11100768 PMC6148664

